# Neutrophils with protumor potential could efficiently suppress tumor growth after cytokine priming and in presence of normal NK cells

**DOI:** 10.18632/oncotarget.2181

**Published:** 2014-07-09

**Authors:** Rui Sun, Jing Luo, Dong Li, Yu Shu, Chao Luo, Shan-Shan Wang, Jian Qin, Gui-Mei Zhang, Zuo-Hua Feng

**Affiliations:** ^1^ Department of Biochemistry and Molecular Biology, Tongji Medical College, Huazhong University of Science & Technology, Wuhan, The People's Republic of China

**Keywords:** neutrophils, protumor potential, priming, cytokines, NK cells

## Abstract

In tumor-bearing state, the function of neutrophils is converted from tumor-suppressing to tumor-promoting. Here we report that priming with IFN-γ and TNF-α could convert the potential of neutrophils from tumor-promoting to tumor-suppressing. The neutrophils with protumor potential have not lost their responsiveness to IFN-γ and TNF-α. After priming with IFN-γ and TNF-α, the potential of the neutrophils to express *Bv8* and *Mmp9* genes was reduced. Conversely, the tumor-promotional neutrophils recovered the expression of *Rab27a* and *Trail*, resumed the activation levels of PI3K and p38 MAPK pathways in response to stimuli, and expressed higher levels of IL-18 and NK-activating ligands such as RAE-1, MULT-1, and H60. Therefore, the anti-tumor function of the neutrophils was augmented, including the cytotoxicity to tumor cells, the capability of degranulation, and the capacity to activate NK cells. Since the function of NK cells is impaired in tumor-bearing state, the administration of normal NK cells could significantly augment the efficiency of tumor therapy based on neutrophil priming. These findings highlight the reversibility of neutrophil function in tumor-bearing state, and suggest that neutrophil priming by IFN-γ/TNF-α might be a potential approach to eliminate residual tumor cells in comprehensive strategy for tumor therapy.

## INTRODUCTION

Polymorphonuclear leukocytes (PMNs or neutrophils) make up a significant portion of the inflammatory cell infiltrate found in various human cancers and murine models [1]. In recent years there is substantial evidence to show significant pro-tumor actions of neutrophils [2-7]. The increased peripheral blood neutrophil counts and neutrophil-to-lymphocyte ratio have been associated with poor clinical outcomes and short overall survival [8-10]. These findings suggested that neutrophils might be a potential target for tumor therapy. However, although the depletion of neutrophils showed antitumor effect [11-13], it is still difficult to consider neutrophil depletion as an approach for tumor therapy due to the important physiological function of neutrophils. Therefore, a new approach for tumor therapy by targeting neutrophils might be, instead of depleting neutrophils, the conversion of neutrophil function from tumor-promoting to tumor-suppressing.

Neutrophils could promote tumor progression by producing proangiogenic factors such as Bv8 [4, 5], and MMP-9 [2, 3]. On the other hand, however, neutrophils could release TRAIL, myeloperoxidase (MPO) and neutrophil elastase (NE), which not only suppress angiogenesis and induce vascular disruption, but also induce apoptosis and inhibit the proliferation of tumor cells [6, 14]. Therefore, increasing the expression of Bv8 and MMP-9 and reducing the expression or release of TRAIL, MPO and NE might be crucial for the protumor function of neutrophils. Conversely modulating the expression and/or release of these factors might be crucial for the conversion of neutrophil function from tumor-promoting to tumor-suppressing. It is known that the priming of neutrophils by inflammatory cytokines such as IFN-γ and TNF-α could enhance the capability of neutrophils to degranulate in response to suitable stimuli, which is the main way for neutrophils to release TRAIL, MPO and NE [15-17]. However, so far it is unknown whether the neutrophils with protumor potential could be remodeled by priming with these cytokines.

In tumor-bearing state, G-CSF and IL-6 cooperated to enhance the potential of neutrophils to express Bv8 and MMP-9, and decrease the ability of neutrophils to release TRAIL, MPO and NE [7]. Consistently, the increased levels of serum G-CSF and IL-6 have been associated with poor prognosis in different types of cancer [18-22]. Therefore, in this study we investigated whether IFN-γ and TNF-α could modulate the expression and/or release of the above-mentioned factors by neutrophils with protumor potential (or G-CSF/IL-6-primed neutrophils), thus reversing the function of the neutrophils. Our data showed that protumor neutrophils can be remodeled by IFN-γ and TNF-α, even in the presence of G-CSF/IL-6, resulting in the conversion of neutrophil function from tumor-promoting to tumor-suppressing. Our data suggest that neutrophils might be indeed a promising target for tumor therapy.

## RESULTS

### Tumor-promoting neutrophils could be remodeled in inflammatory environment

**Figure 1:**
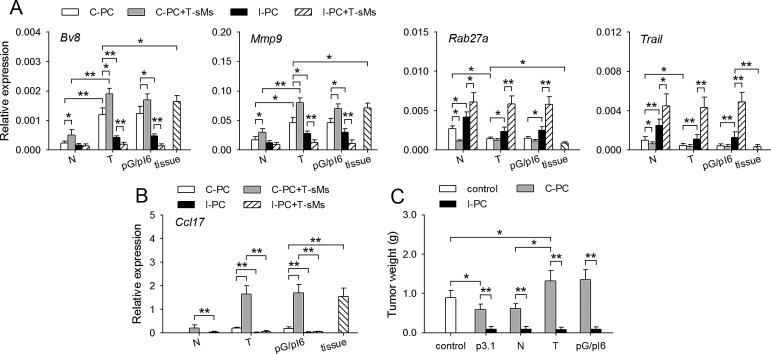
The function of protumor neutrophils is remodeled in inflammatory environment (A, B) Neutrophils were isolated from C-PC and I-PC of naive mice (N), tumor-bearing mice (T), pG/pI6-mice (pG/pI6), or from tumor tissues (tissue). The neutrophils from C-PC and I-PC were unstimulated or stimulated with T-sMs (0.5 mg/ml) for 12 h. The expression of *Bv8*, *Mmp9, Rab27a*, and *Trail* was detected by real-time RT-PCR (A). The expression of *Ccl17* in the neutrophils from C-PC and I-PC was detected by real-time RT-PCR (B). (C) Neutrophils were isolated from C-PC and I-PC of naive mice, tumor-bearing mice, and pG/pI6-mice. The neutrophils were used for co-inoculation with H22 cells to naive mice. Data are pooled from four independent experiments with a total of eight samples in each group. *p < 0.05, **p < 0.01.

In tumor-bearing state, G-CSF/IL-6 could induce the protumor potential of neutrophils in bone marrow [7]. To explore whether the function of neutrophils could be remodeled, we recruited neutrophils to the non-inflammatory and inflammatory peritoneal cavity of naive mice, tumor-bearing mice and the mice with in vivo expression of G-CSF and IL-6 (pG/pI6-mice). When neutrophils were recruited to peritoneal cavity with CXCL1-expressing cells (C-PC, non-inflammatory) [7], the neutrophils from tumor-bearing mice and pG/pI6-mice showed a protumor phenotype, characterized by higher expression of *Bv8* and *Mmp9* as well as lower expression of *Rab27a* and *Trail* [7]. The stimulation of these neutrophils with soluble molecules from tumor (T-sMs) further modulated the expression of these genes, resulting in the same expression pattern of these genes as that of the neutrophils from tumor tissues (Figure 1A). However, when neutrophils were recruited to inflammatory peritoneal cavity (I-PC), the expression pattern of these genes was reversed. The stimulation with T-sMs resulted in the same gene expression pattern as that of the neutrophils from naive mice (Figure 1A).

When the neutrophils were isolated from tumor-bearing mice and pG/pI6-mice, the expression of *Ccl17* gene was induced by T-sMs in the neutrophils from C-PC, but not the neutrophils from I-PC (Figure 1B), suggesting that the phenotype of the neutrophils might be switched to antitumorigenic in inflammatory environment, since *Ccl17* is expressed in the neutrophils with a protumorigenic phenotype but not those with antitumorigenic phenotype [12, 23, 24]. To further confirm this, we tested the function of the neutrophils from C-PC and I-PC in co-inoculation test. The neutrophils from C-PC of tumor-bearing mice and pG/pI6-mice promoted tumor growth, whereas the neutrophils from I-PC showed a strong inhibitory effect on tumor growth (Figure 1C). Taken together, these results suggest that the potential of neutrophils could be converted from protumor to antitumor in inflammatory environment.

### IFN-γ and TNF-α could induce the conversion of neutrophil function

**Figure 2:**
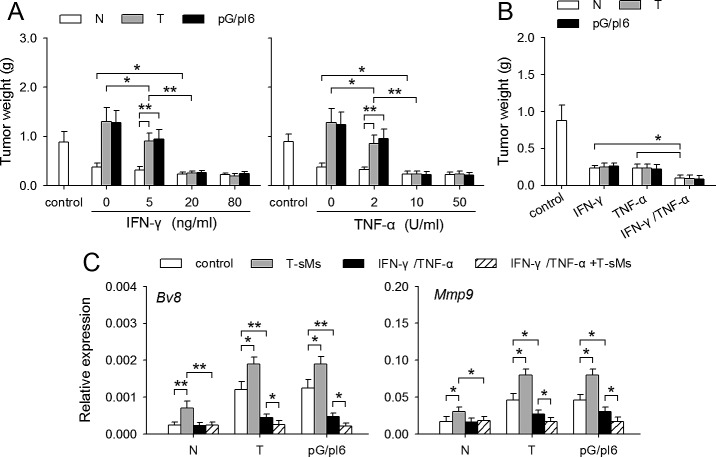
IFN-γ and TNF-α reverse the effect of protumor neutrophils on tumor growth Neutrophils were isolated from the C-PC of naive mice, tumor-bearing mice and pG/pI6-mice. (A) Neutrophils were treated with IFN-γ or TNF-α at the indicated concentration for 6 h and then used for co-inoculation with H22 cells to naive mice. (B, C) Neutrophils were treated with IFN-γ (20 ng/ml) and/or TNF-α (10 U/ml) for 6 h. The cells were then used for co-inoculation with H22 cells to naive mice (B). Or the cells were stimulated with T-sMs for 12 h, and then used for detecting the expression of *Bv8* and *Mmp9* by real-time RT-PCR (C). Data are pooled from four independent experiments with a total of eight samples in each group. *p < 0.05, **p < 0.01.

To explore the mechanisms underlying the conversion of neutrophil function in inflammatory environment, we focused on the inflammatory cytokine IFN-γ and TNF-α which were produced in I-PC, but not in C-PC (Supplemental Figure S1). The neutrophils from C-PC of different mice were treated with IFN-γ and/or TNF-α. After the treatment, the neutrophils from both tumor-bearing mice and pG/pI6-mice showed anti-tumor function in co-inoculation test (Figure 2A). The co-stimulation with IFN-γ and TNF-α was more efficient in promoting anti-tumor function of neutrophils (Figure 2B). We then analyzed whether IFN-γ and TNF-α could reverse the expression of *Bv8* and *Mmp9* in neutrophils. IFN-γ, but not TNF-α, could significantly reduce the expression of *Bv8* and *Mmp9* in the neutrophils from tumor-bearing mice and pG/pI6-mice, and abrogate the promoting effect of T-sMs on the expression of *Bv8* and *Mmp9* (Supplemental Figure S2). The same effect was observed when the neutrophils were treated with IFN-γ in combination with TNF-α (Figure 2C). These results suggest that IFN-γ and TNF-α could efficiently induce the conversion of neutrophil function, although only IFN-γ has an inhibitory effect on the expression of *Bv8* and *Mmp9*.

### IFN-γ and TNF-α resume the antitumor potential of neutrophils

**Figure 3:**
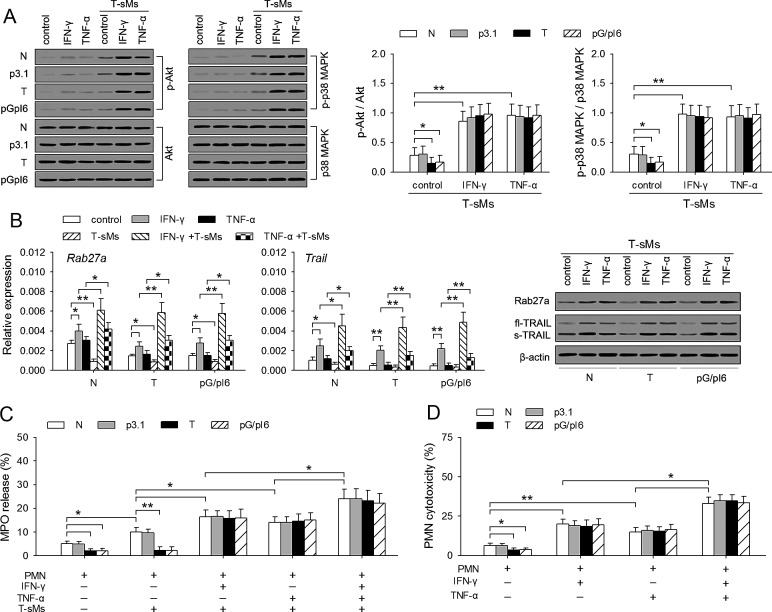
IFN-γ and TNF-α resume the antitumor potential of neutrophils Neutrophils were isolated from C-PC of naive mice, tumor-bearing mice, and pG/pI6-mice. After the treatment with IFN-γ (20 ng/ml) or TNF-α (10 U/ml) for 6 h, the neutrophils were stimulated with T-sMs (0.5 mg/ml) for 0.5 h (A, C) or 12 h (B), or without further stimulation (D). (A) The phospho-Akt, Akt, phospho-p38 MAPK, and p38 MAPK were detected by Western blot (*left*). The ratios of phospho-Akt to Akt (p-Akt/Akt) and phospho-p38 MAPK to p38 MAPK (p-p38 MAPK/p38 MAPK) were calculated after densitometric analysis (*right*). (B) The expression of *Rab27a* and *Trail* was detected by real-time RT-PCR and Western blot. (C) MPO release was detected. (D) The cytotoxicity of neutrophils to H22 tumor cells was detected as described in Methods. Data are representative of three independent experiments (A, left; B, right), or pooled from three independent experiments with a total of eight samples in each group (A, right; B, left; C; D). *p < 0.05, **p < 0.01.

We next investigated whether IFN-γ and TNF-α could resume the antitumor potential of the neutrophils from tumor-bearing mice and pG/pI6-mice. In tumor-bearing state, the capability of neutrophils to degranulate is attenuated due to the inhibitory effect of G-CSF/IL-6 on activation of PI3K and p38 MAPK pathways and the expression of Rab27a [7]. After priming with IFN-γ and TNF-α, the activation of PI3K and p38 MAPK pathways in the neutrophils from tumor-bearing mice and pG/pI6-mice in response to T-sMs was increased to the levels similar to those in the neutrophils from naive mice (Figure 3A). Moreover, the treatment with IFN-γ increased the expression of *Rab27a* gene in neutrophils from different mice (Figure 3B). T-sMs down-regulated *Rab27a* expression in the un-primed neutrophils. However, if the neutrophils were pre-treated with IFN-γ or TNF-α, T-sMs increased *Rab27a* expression in the neutrophils from tumor-bearing mice and pG/pI6-mice to the similar level of that in the neutrophils from naive mice (Figure 3B). The expression of *Trail* and the production of soluble TRAIL were similarly modulated (Figure 3B).

The treatment with IFN-γ or TNF-α did not influence the spontaneous degranulation of neutrophils (Supplemental Figure S3), but augmented the induced degranulation by neutrophils in response to T-sMs, evaluated by the release of MPO (Figure 3C). The release of NE and soluble TRAIL was also increased (data not shown). Consistent with the release of these factors, the cytotoxicity of neutrophils to tumor cells was augmented by priming with IFN-γ and TNF-α (Figure 3D; Supplemental Figure S4). Intriguingly, the neutrophils from C-PC of tumor-bearing mice and pG/pI6-mice showed the cytotoxicity similar to that of the neutrophils from naive mice after priming. Taken together, these results demonstrate that the response of protumor neutrophils to T-sMs was reversed by priming with IFN-γ and TNF-α, thus resulting in the conversion of neutrophil function from tumor-promotional to tumor-suppressing.

### Neutrophil function could be remodeled *in vivo* by local expression of IFN-γ and TNF-α

We next investigated whether the conversion of neutrophil function could be induced in vivo by local expression of IFN-γ and/or TNF-α. Lower dosages of expression vectors were used for local transfection to express IFN-γ, soluble TNF-α (sTNF-α), and neutrophil chemokine CXCL1. When naive mice were inoculated with tumor cells, IFN-γ and sTNF-α cooperated with CXCL1 to more efficiently suppress tumor growth (Supplemental Figure S5). Although TNF-α could suppress tumor growth by directly inducing the apoptosis of tumor cells via TNFR-1 [25], the neutrophils recruited to local tissue were important for the anti-tumor effect of TNF-α. The anti-tumor effect of TNF-α and/or IFN-γ was augmented by neutrophil chemokine CXCL1, and was attenuated by depleting neutrophils (Supplemental Figure S5B and S5C).

**Figure 4:**
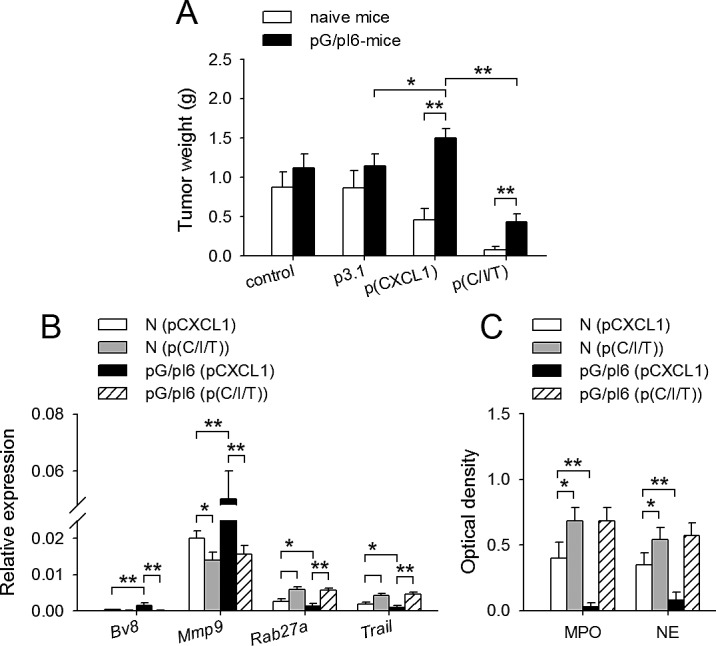
Neutrophil function could be remodeled *in vivo* by IFN-γ and TNF-α Naive mice and pG/pI6-mice were inoculated with H22 cells and treated with pCXCL1 (20 μg) or pCXCL1/pIFN-γ(40 μg)/psTNF-α (10 μg) as described in Methods. (A) Tumors were dissected and weighed on d11 after inoculation. (B) Neutrophils were isolated from tumor tissue on d7 after inoculation. The expression of *Bv8*, *Mmp9*, *Rab27a*, and *Trail* was detected by real-time RT-PCR. (C) MPO and NE in the tumor tissues were detected as described in Methods on d7 after inoculation. Data are pooled from four independent experiments with a total of eight mice in each group. *p < 0.05, **p < 0.01.

When pG/pI6-mice were inoculated with tumor cells, CXCL1 promoted tumor growth. IFN-γ and TNF-α reversed the effect of CXCL1, but were less efficient in suppressing tumor growth (Figure 4A). We therefore investigated whether G-CSF and IL-6 might antagonize the priming of neutrophils by IFN-γ and TNF-α. The results showed that G-CSF/IL-6 could not antagonize the priming effect of IFN-γ/TNF-α, evaluated by the activation of signaling pathways, MPO release, and tumor-suppressing effect of neutrophils (Supplemental Figure S6).

We then compared the neutrophils from the tissues at tumor inoculation site after local expression of CXCL1/IFN-γ/sTNF-α in naive mice and pG/pI6-mice. The results showed that IFN-γ/sTNF-α indeed modulated the expression of *Bv8*, *Mmp9, Rab27a*, and *Trail* in neutrophils in vivo (Figure 4B), resulting in similar expression pattern of these genes in the neutrophils of naive mice and pG/pI6-mice. Moreover, MPO and NE in the local tissues of pG/pI6-mice reached similar levels to those in naive mice after treatment (Figure 4C). These results suggested that the neutrophils were similarly primed by IFN-γ/sTNF-α in pG/pI6-mice and naive mice.

### NK activating capability of neutrophils is efficiently induced by IFN-γ and TNF-α

**Figure 5:**
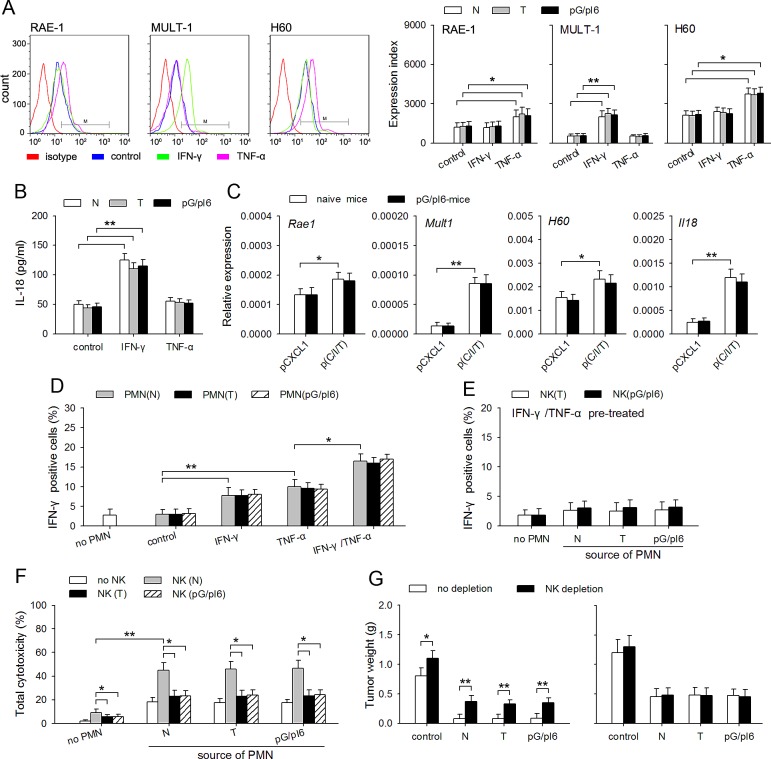
NK activating capability of neutrophils is efficiently induced by IFN-γ and TNF-α (A, B) Neutrophils were isolated from C-PC of naive mice, tumor-bearing mice and pG/pI6-mice. The cells were stimulated with IFN-γ (20 ng/ml) or TNF-α (10 U/ml) for 12 h (A) or 18 h (B). (A) The protein level of RAE-1, MULT-1, and H60 was detected by flow cytometry (a, *left*). Expression index was calculated as described in Methods (a, *right*). (B) IL-18 in the culture supernatant was detected by ELISA. (C) The neutrophils were isolated from tumor tissues after treatment with pCXCL1(20 μg)/pIFN-γ(40 μg)/psTNF-α(10 μg). The expression of *Rae1*, *Mult1*, *H60*, *Il18* was detected by real-time RT-PCR. (D, E) Neutrophils were isolated from C-PC of naive mice, tumor-bearing mice and pG/pI6-mice. After the treatment with IFN-γ and/or TNF-α for 12 h, and the removal of IFN-γ and TNF-α by washing with PBS, the neutrophils were co-cultured with the NK cells isolated from naive mice (D) or tumor-bearing mice and pG/pI6-mice (E) for 12 h. IFN-γ-expressing NK cells were detected by flow cytometry as described in Methods. (F) Neutrophils (from I-PC) and NK cells (from spleen) were isolated from naive mice, tumor-bearing mice and pG/pI6-mice. Neutrophils (2×10^6^) and NK cells (1×10^5^) were co-cultured for 12 h, and then incubated with H22 cells (1×10^5^) for the assay of cytotoxicity as described in Methods. (G) The neutrophils from I-PC of naive mice, tumor-bearing mice and pG/pI6-mice were used for co-inoculation with H22 cells to naive mice (left) or pG/pI6-mice (right). NK cells were depleted in vivo as indicated (NK depletion). Data are representative of three independent experiments (A, left), or pooled from three independent experiments with a total of eight samples in each group (A, right; B-G). *p < 0.05, **p < 0.01.

The data in Figure 4 showed that the neutrophils in pG/pI6-mice and naive mice were similarly primed by IFN-γ/TNF-α, but CXCL1/IFN-γ/TNF-α was less efficient in suppressing tumor growth in pG/pI6-mice, suggesting that the release of anti-tumor factors such as TRAIL, MPO, and NE might only partially contribute to the anti-tumor effect of the primed neutrophils. Since activating NK cells is another aspect of neutrophils' function [26], we therefore wondered whether G-CSF and IL-6 might influence the NK-activating capacity of neutrophils. To clarify this, we first analyzed the expression of NK-activating ligands in neutrophils, including RAE-1, MULT-1, and H60 [27]. The results showed that TNF-α could up-regulate the expression of RAE-1 and H60, and that IFN-γ promoted the expression of MULT-1 (Figure 5A; Supplemental Figure S7). Moreover, IFN-γ up-regulated the expression of IL-18 (Figure 5B; Supplemental Figure S7) which can promote the activity of NK cells [28]. There was no difference between the neutrophils from naive mice and pG/pI6-mice, which was also confirmed by analyzing the neutrophils isolated from the tissues at tumor inoculation site (Figure 5C). Moreover, the primed neutrophils from different mice could similarly express higher level of CCL3, a chemokine for recruiting NK cells [29], and promote the infiltration of NK cells in local tissue (supplemental Figure S8).

After priming with IFN-γ and/or TNF-α, the neutrophils from different sources could similarly induce the production of IFN-γ by the NK cells from naive mice (Figure 5D; Supplemental Figure S9), but not the NK cells from tumor-bearing mice and pG/pI6-mice (Figure 5E). The NK cells from tumor-bearing mice and pG/pI6-mice could not cooperate with the primed neutrophils to produce stronger cytotoxicity to H22 tumor cells (Figure 5F; Supplemental Figure S10). Consistently, splenocytes from tumor-bearing mice and pG/pI6-mice showed lower cytotoxicity to YAC-1 cells (Supplemental Figure S11). Moreover, depleting NK cells significantly attenuated the anti-tumor effect of the primed neutrophils in naive mice, but not in pG/pI6-mice (Figure 5G). Taken together, these results suggest that priming with IFN-γ and TNF-α could augment the NK-activating capability of the neutrophils from different sources, but the impairment of NK cell function could attenuate the antitumor effect of the neutrophils.

### Normal NK cells are required for better efficacy of therapeutic approach based on neutrophil priming

**Figure 6:**
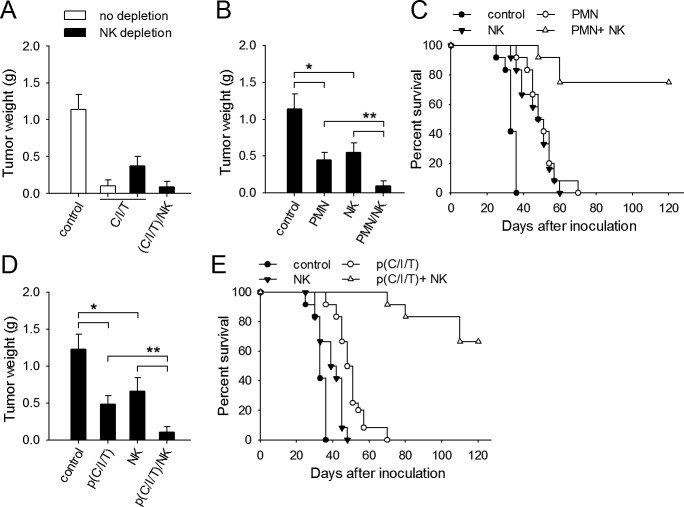
Normal NK cells are required for better efficacy of therapeutic approach based on neutrophil priming (A) Naive mice with or without NK depletion were inoculated with H22 cells. The mice were treated with pCXCL1/pIFN-γ/psTNF-α with or without the injection of NK cells from naive mice. (B, C) pG/pI6-mice were inoculated with H22 cells together with neutrophils from I-PC of pG/pI6-mice and/or purified NK cells from naive mice. (D, E) pG/pI6-mice were inoculated with H22 cells, and then treated with pCXCL1/pIFN-γ/psTNF-α. Purified NK cells from naive mice were injected to the inoculation site as described in Methods. Tumors were dissected and weighed on d11 after inoculation (A, B, D). The survival rate of mice in each group was recorded in parallel experiments (C, E). Data are pooled from four independent experiments with a total of eight mice in each group (A, B, D), or the combination of two independent experiments with a total of twelve mice in each group (C, E). *p < 0.05, **p < 0.01.

To further confirm that normal functional NK cells are in favor of the therapeutic strategy based on the conversion of neutrophil function, we further tested the therapeutic effect of local expression of CXCL1/IFN-γ/sTNF-α in the presence or absence of normal NK cells. When naive mice were inoculated with tumor cells, the therapeutic effect of CXCL1/IFN-γ/sTNF-α was attenuated by depleting NK cells, but recovered by administrating NK cells (Figure 6A). When pG/pI6-mice were inoculated with tumor cells, the co-inoculation with the primed neutrophils and normal NK cells was more efficient in suppressing tumor growth, evaluated by the tumor weight and the survival of mice (Figure 6B, C).

We then inoculated pG/pI6-mice with H22 tumor cells, and treated the mice by local expression of CXCL1/IFN-γ/sTNF-α and the injection of normal NK cells. The administration of NK cells significantly promoted the therapeutic effect of the combined gene therapy (Figure 6D, E). These results suggest that the NK cells with normal function could indeed enhance the efficacy of therapeutic approach based on priming of neutrophils by IFN-γ and TNF-α.

## DISCUSSION

Our data in this study showed that priming with IFN-γ and TNF-α could convert the function of neutrophils from tumor-promoting to tumor-suppressing. The primed neutrophils not only resumed their potential to release antitumor factors in response to T-sMs, but also activated NK cells to more efficiently suppress tumor growth. Therefore, although the depletion of neutrophils showed antitumor effect [11-13], reversing the protumor potential of neutrophil might be a more suitable approach in tumor therapy because of the important physiological function of neutrophils. Importantly, the simultaneous modulation of NK cell function would be necessary for better anti-tumor efficiency of neutrophils after the conversion of their function.

The neutrophils in tumor may have differential states of activation/differentiation, thus taking an antitumorigenic (“N1”) versus a protumorigenic (“N2”) phenotype [12]. Interestingly, the stimulation of neutrophils by T-sMs could induce either a “N1” phenotype or “N2” phenotype of neutrophils, which was determined by the responsiveness of the neutrophils to T-sMs as shown by our previous [7] and present studies. T-sMs may contain most, if not all of, signal molecules in tumor which might have different effects on neutrophils. Our data suggest that the final phenotype of neutrophils in tumor might be actually determined by the responsiveness of the neutrophils to T-sMs. In tumor-bearing state, the protumor potential of neutrophils is induced in bone marrow, which is mediated by G-CSF/IL-6 [7]. A “N2” phenotype of these neutrophils could be induced by further stimulation with T-sMs. However, when G-CSF/IL-6-primed neutrophils were further primed by IFN-γ/TNF-α, the stimulation with same T-sMs could induce a “N1” phenotype of neutrophils. Importantly, our data demonstrate that the protumor potential of neutrophils in tumor-bearing state and their reactivity to T-sMs are reversible. Neutrophils are short-lived leukocytes. Therefore, altering the responsiveness of newly-recruited neutrophils to T-sMs might be sufficient for switching the effect of neutrophils on tumor from promotional to suppressive.

Priming by cytokines could augment the responses of neutrophils to subsequent stimulation [17, 30, 31]. Accordingly, priming with either G-CSF/IL-6 or IFN-γ/TNF-α could augment the responsiveness of neutrophils to T-sMs, resulting in different phenotypes. In tumor-bearing state, G-CSF/IL-6 could endow neutrophils with protumor potential by inducing the enhanced activation of STAT3 and attenuating the activation of PI3K and p38 MAPK pathways in response to T-sMs [7]. The enhanced activation of STAT3 is crucial for modulating the expression of *Bv8*, *Mmp9*, *Rab27a*, *Trail* [7]. IFN-γ and TNF-α have the potential to antagonize the effect of STAT3. IFN-γ antagonizes the effect of STAT3 by activating STAT1 and SOCS3 [32, 33]. TNF-α promotes STAT1 activation by increasing serine phosphorylation of STAT1 [34], and also negatively modulates the effect of STAT3 by activating PI3K pathway [35, 36]. Nevertheless, it has been unclear whether IFN-γ and TNF-α are able to modulate the response of G-CSF/IL-6-primed neutrophils to T-sMs. Intriguingly, our data showed that G-CSF/IL-6-primed neutrophils have not lost their responsiveness to IFN-γ and TNF-α. The treatment with IFN-γ/TNF-α not only reversed STAT3-mediated modulatory effects on gene expression, but also abolished the negative effect of G-CSF/IL-6 on the activation of PI3K and p38 MAPK pathways. Importantly, when G-CSF, IL-6, IFN-γ, and TNF-α were simultaneously existent, IFN-γ and TNF-α showed a dominant effect in neutrophil priming. Therefore, the treatment with IFN-γ/TNF-α enabled T-sMs to induce anti-tumor function of the neutrophils by increasing the production and/or release of MPO, NE, and TRAIL, and reducing the expression of *Bv8* and *Mmp9* genes.

NK cells can suppress tumor by producing apoptosis-inducing ligands (TRAIL, FasL) and cytotoxic granules (perforin, granzyme B) [37]. In addition to reversing the reactivity of neutrophils to T-sMs, priming by IFN-γ and TNF-α could enhance the capability of neutrophils to activate NK cells. Same as the neutrophils from naive mice, the tumor-promoting neutrophils could produce more IL-18, and express higher levels of NK-activating ligands RAE-1, MULT-1, and H60 after priming by IFN-γ and TNF-α. These neutrophils were capable of activating normal NK cells. However, the primed neutrophils were unable to activate NK cells in tumor-bearing mice and pG/pI6-mice. The impairment of NK cell function restricted the anti-tumor effect of the primed neutrophils. The impaired function of NK cells has been observed in the patients with different types of tumors and murine tumor models [38, 39]. Our data in this study showed that the increased G-CSF and IL-6 in vivo might be one of reasons for the impairment of NK cell function. This finding is also supported by another report that both cytotoxicity and reactivity of NK cells in bone marrow was declined by G-CSF [40]. In this study, we were unable to explain the exact mechanism underlying the impairment of NK cell function by the increased expression of G-CSF and IL-6 in vivo. Nevertheless, it has been found that the altered function of bone-marrow neutrophils could influence the maturation of NK cells [41, 42], suggesting that the impairment of NK cell function might be related to the alteration of neutrophil function in bone-marrow by G-CSF and IL-6.

Although our data suggest that IFN-γ and TNF-α could efficiently reverse the function of neutrophils, the systemic administration of IFN-γ and TNF-α has not been very effective in the treatment of tumors, probably due to the systemic toxic side effect produced by these broad-acting treatments [43, 44]. Increasing the expression of IFN-γ and TNF-α by local transfection of expression vectors might be an alternative approach for neutrophil priming. Importantly, the combinatorial utilization of IFN-γ and TNF-α might reduce the requirement for higher levels of these cytokines in vivo, which might minimize the side effect. In addition, further study on the mechanisms underlying the impairment of NK cell function is required, since the NK cells with normal function could significantly augment the antitumor effect of neutrophils. In summary, the findings in this study not only highlight the reversibility of neutrophil function in tumor-bearing state, but also suggest that neutrophil priming by IFN-γ/TNF-α might be a potential approach to eliminate residual tumor cells in comprehensive strategy for tumor therapy.

## MATERIALS AND METHODS

### Animals and cell lines

BALB/c mice, 6 to 8-week-old, were purchased from Center of Medical Experimental Animals of Hubei Province (Wuhan, China) for studies approved by the Animal Care and Use Committee of Tongji Medical College. YAC-1 lymphoma cell line and BALB/c background H22 hepatocarcinoma cell line were purchased from China Center for Type Culture Collection (Wuhan, China) and cultured according to their guidelines.

### Reagents and Plasmids

Murine TNF-α, IFN-γ, G-CSF and IL-6 were purchased from PeproTech (Rocky Hill, NJ). Murine IL-2 was purchased from Millipore (Billerica, MA). PE-conjugated anti-mouse IFN-γ, PE-conjugated anti-mouse MULT-1 were purchased from eBioscience (San Diego, CA). PE-conjugated anti-mouse H60 and PE-conjugated anti-panRAE-1 were purchased from R&D Systems. Anti-asialo-GM1 mAb was purchased from Biolegend. Anti-Ly6G mAb was purchased from BioExpress (clone 1A8).

Plasmids pG, pI6, pCXCL1, and pIFN-γ are expression vectors carrying the cDNA encoding murine G-CSF, IL-6, CXCL1, and IFN-γ, respectively. Plasmid psTNF-α is the expression vector carrying the cDNA encoding IFN-β signal peptide and extracellular domain of TNF-α. These plasmids were constructed by the insertion of cDNA into plasmid pcDNA3.1 (Invitrogen, Carlsbad, CA) in our laboratory. All of the vectors were identified by in vivo expression (Ref 7 and Supplemental Figure S12).

### Recruitment of neutrophils to peritoneal cavity

To recruit neutrophils to non-inflammatory peritoneal cavity, CXCL1-expressing hepatocytes were injected to peritoneal cavity of mice as described previously [7]. The peritoneal cells were harvested 12 h later. To recruit neutrophils to inflammatory peritoneal cavity, 3 ml of 3% Thioglycolate Broth Solution (containing 10^9^ *E. coli* bacteria) was injected into the peritoneal cavity of mice, followed by one more injection 12 h later. Then, the peritoneal cells were harvested 2 h after the second injection.

### Isolation of neutrophils

The neutrophils were isolated from peritoneal cells or from tissues as described previously [7]. Briefly, to isolate neutrophils from peritoneal cells, the cells were washed once in HBSS, layered over a Percoll gradient 54%/64%/80%, and centrifuged at 1060 × *g* for 30 min. The dense band at 64%/80% interface was collected as neutrophil fraction. To isolate neutrophils from tissues, single cell suspensions were prepared by digesting the tissues with collagenase, hyaluronidase, and DNase. Neutrophils were isolated by using PE-anti-Ly6G antibody (eBioscience), magnetic microbeads, and MiniMACS columns (Miltenyi Biotec) according to manufacturer's protocol. The isolated cells were >90% neutrophils as assessed by flow cytometric analysis (Supplemental Figure S13A).

### Analysis of gene expression by real-time RT-PCR

Total RNA was extracted from cells with TRIzol reagent (Invitrogen). The relative quantity of mRNA was determined by real-time RT-PCR according to MIQE guideline [45]. *Gapdh*, *Hprt*, and *Ppia* were chosen as reference genes. The relative expression of gene was calculated using GeNorm software. The primer sequences were as follows: *Gapdh*, sense 5′-ATGTTCCAGTATGACTCCACTCAC-3′, antisense 5′-GACACCAGTAGACTCCACGA CATA-3′; *Hprt*, sense 5′-GGGCTATAAGTTCTTTGCTGACCT-3′, antisense 5′-CCCGTTGACTGATCATTACAGTAG-3′; *Ppia*, sense 5′-CTATAAGGGTTCCTCCTTTCACAG-3′, antisense 5′-CAGGACCTGTATGCTTTAGGATG-3′; *Bv8*, sense 5′-TGCTACTTCTGCTGCTACC-3′, antisense 5′-CCGCACTGAGAGTCCTTGTC-3′; *Mmp9*, sense, 5′-TGCCCAGCGACCACAACTC-3′, antisense 5′-CGGACCCGAAGCGGACATT-3′; *Ccl17*, sense 5′-GCTGAGGCATTTGGAGAC-3′; antisense 5′-GAGGAAGGCTTTATTCCG-3′; *Rab27a*, sense 5′-CGTGCCTTCCAGCGTTGTTC-3′, antisense 5′-TCGCCTGCCTCTGCTTCTCA-3′; *Trail*, sense 5′-TACTGGGATCACTCGGAGAAG-3′, antisense 5′-ACGTGGTTGAGAAATGAATGCC-3′; *Rae1*, sense 5′-CATGGGCGAATGATGTT-3′, antisense 5′-TTGG AGGGTTGATGTAGTGA-3′; *Mult1*, sense 5′-GGATACGCCATTGTTAGCCT-3′, antisense 5′-TGCAGTCGCCCTCTGTAGT-3′; *H60*, sense 5′-GTCACTGCCTCAACAAATCGTC-3′, antisense 5′-CAGCATACACCAAGCGAATACC-3′; *Il18*, sense 5′-AATCTGTAATGTTCACTCTCACT-3′, antisense 5′-TACTCTATAAATCATGCA GCCTCGG-3′.

### *In vivo* gene transfection

Plasmids were prepared and analyzed as described previously [46]. The mice received the injection of plasmid DNA (in 100 μl of saline) into the muscle tissue (i.m. injection) at tumor inoculation site or without tumor inoculation as indicated.

### Animal experiments and treatment protocol

To acquire the mice with in vivo expression of G-CSF/IL-6 (pG/pI6-mice), mice received i.m. injection of pG and pI6 plasmids (100 μg of each per injection) in left hind thigh, once every 2 days for 4 times [7]. Plasmid pcDNA3.1 was used as a control. The mice were used for further experiments on d10 after the first injection of plasmid. Naive mice and the mice receiving pcDNA3.1 injection were labeled by N and p3.1 respectively.

In co-inoculation test, mice were inoculated intramuscularly in the right hind thigh with 1×10^5^ H22 cells, mixed with 1×10^6^ neutrophils and/or 1×10^5^ NK cells. For treatment experiments, the mice received i.m. injection of the indicated plasmid DNA (in 100 μl saline) every 2 days starting from d1 after inoculation. Saline and pcDNA3.1 were used as controls. In some experiments, 1×10^5^ NK or an equal volume of PBS (50 μl) was injected to the inoculation site on d1, d4, and d7 after inoculation. Tumors were dissected and weighed on d11 after inoculation. Or the mice were monitored every 3 days for recording the survival.

To isolate neutrophils from tumor-bearing mice, mice were inoculated intramuscularly with 1×10^5^ H22 cells. The mice were used for isolation of neutrophils on d10 after inoculation. In some experiments, neutrophils were recruited to the tissue at inoculation site by local expression of CXCL1 [7]. The tissues at inoculation site were used for isolation of neutrophils on d7 after inoculation.

### Assay of MPO and NE

The MPO and NE in tissues, and MPO release by neutrophils, were detected as described previously [7]. To induce the release of azurophilic granules, neutrophils were stimulated for 30 min with T-sMs (0.5 mg/ml). Neutrophil degranulation was determined by detecting the release of MPO. Percentage of MPO release was calculated as described previously [47]. For the detection of MPO and NE in tissues, soluble interstitial molecules in the tissues at inoculation sites were prepared on d7 after tumor inoculation. The mixture of soluble molecules from tissues was prepared by digesting the tissues with collagenase and removing debris by centrifugation. To detect MPO and NE in tissues, soluble interstitial molecules prepared from tissues were diluted to 0.5 mg/ml (for MPO) and 2.5 mg/ml (for NE) of proteins. The activities of MPO and NE were measured as described previously [48] and expressed as the values of OD (OD_450_ for MPO and OD_405_ for NE).

### ELISA assay

Cell-free supernatants from neutrophils were harvested after 18-h culture. IL-18 in the supernatants was quantified using ELISA kit (R&D Systems, Minneapolis, MN).

### Isolation of NK cells

NK cells were isolated from spleen cells using anti-DX5 magnetic microbeads, and MiniMACS columns (Miltenyi Biotec). The isolated cells were further identified by staining with FITC-conjugated anti-CD335 (NKp46) and PE-conjugated anti-mouse CD3e. The isolated cells were >82% NK cells as assessed by flow cytometric analysis (Supplemental Figure S13B).

### Cytotoxicity assay

The cytotoxicity of neutrophils and NK cells to tumor cells was determined as described previously [49, 50]. Briefly, CFSE-labeled H22 cells were incubated with the effector cells (neutrophils and/or NK cells) at 37 °C for 6 h. The cells were then stained with allophycocyanin-Annexin-V (BD Biosciences, San Diego, CA) and analyzed by flow cytometry. The cytotoxicity of NK cells to YAC-1 cells was expressed as the percentage of CFSE^+^Annexin-V^+^ cells in CFSE^+^ cells, which was calculated by the following formula: cytotoxicity % = (CFSE^+^Annexin-V^+^ cells/CFSE^+^ cells in the mixture of YAC-1 cells and effector cells) – (Annexin-V^+^CFSE^+^ cells/CFSE^+^ cells in control YAC-1 cells).

### Western blot assay

Western blot assay was done as described previously [51]. Antibodies were purchased from Cell Signaling Technology (Beverly, MA) and Santa Cruz Biotechnology (Santa Cruz, CA).

### *In vivo* depletion of neutrophils and NK cells

To deplete neutrophils, anti-Ly6G antibody was used [52]. Mice receive an i.p. injection of anti-Ly6G at a dose of 300 μg in 500 μl PBS on -d1, d1, d4 and d7 of tumor inoculation [53]. To deplete NK cells, anti-asialo-GM1 antibody was used [54]. Each mouse received intraperitoneal injection of 50 μg of anti-asialo-GM1 Ab in 200 μl PBS on -d3, -d1, and d2 of tumor inoculation. The depletion of neutrophils and NK cells was identified by Flow cytometric analysis (Supplemental Figure S14).

### Flow cytometric analysis

Neutrophils were stained with PE-conjugated anti-mouse panRAE-1, MULT-1, H60 or isotype control IgG1 for flow cytometric analysis as previously described [55]. Briefly, neutrophils were stained with PE-conjugated anti-mouse panRAE-1, PE-conjugated anti-mouse MULT-1, PE-conjugated anti-mouse H60 or isotype control IgG1 for flow cytometric analysis. Parameters were acquired on a FACS Calibur flow cytometer (BD Biosciences) and analyzed with CellQuest software (BD Biosciences). Percent staining was defined as the percentage of cells in the gate (M1) which was set to exclude ~99% of isotype control cells. Expression index was calculated by using the formula: mean fluorescence × percentage of positive cells.

### Preparation of soluble molecules from tumor tissues

Palpable tumors were dissected. The mixture of soluble molecules from tumor (T-sMs) was prepared by digesting the tissues with collagenase and removing debris by centrifugation. The concentration of T-sMs was defined by the concentration of protein, which was determined using Coomassie Bradford reagent (Thermo Fisher Scientific, Rockford, IL) according to the manufacturer's instructions.

### Assay of NK activation

The activation of NK cells was detected by staining for intracellular IFN-γ. NK cells were labeled with CFSE and co-cultured with neutrophils in 96-well U-bottom plate at the ratio of (1:20, NK:PMN) for 12 h. The intracellular IFN-γ was stained with PE-conjugated anti-mouse IFN-γ, and analyzed by flow cytometry.

### Statistical analysis

Results were expressed as mean value ± SD and interpreted by one-way ANOVA. Differences were considered to be statistically significant when *p* < 0.05.

## SUPPLEMENTARY MATERIALS AND METHODS, FIGURES


