# Supplementary material for: Neutrophils with protumor potential could efficiently suppress tumor growth after cytokine priming and in presence of normal NK cells
Source: Oncotarget. 2014 Jul 9;5(24):12621–34. doi: 10.18632/oncotarget.2181 (PMC4350330; doi:10.18632/oncotarget.2181)
Supplement: Supplementary file 1 [file oncotarget-05-12621-s001.pdf]

Neutrophils with protumor potential could efficiently suppress tumor growth after cytokine priming and in presence of normal NK cells

Supplementary Materials and Methods

Reagents

PE-conjugated anti-mouse Ly6G, PE-Cy7-conjugated anti-mouse CD11b, PE-conjugated anti-mouse Ly6C, PE-conjugated anti-mouse Gr-1, FITC-conjugated anti-mouse DX5, FITC-conjugated anti-CD335 (NKp46), PE-conjugated anti-mouse CD3e were purchased from eBioscience (San Diego, CA). Rabbit anti-mouse DX5 (clone EPR5788) was purchased from Abcam.

ELISA assay

The peritoneal cavity of each mouse was rinsed with 5 ml of PBS. After centrifugation, the concentration of IFN-γ and TNF-α in the supernatant was detected by mouse-IFN-γ and mouse-TNF-α ELISA kits (Bender MedSystem, Vienna, Austria) according to the manufacture's instructions.

Flow cytometric analysis

To analyze the effect of neutrophil depletion and NK cell depletion in vivo, mice were sacrificed one day after the second injection of antibody. The heparinized blood was harvested for analysis. 20 μl of whole blood was incubated with PE-anti-mouse Ly6G and PE-Cy7-anti-mouse CD11b for 30 min on ice. RBCs were then lysed. The samples were centrifuged at 350 × *g* for 5 min, and then resuspended in 300 μl of PBS for flow cytometric analysis. Ly6G$^+$CD11b$^+$ cells were considered as neutrophils. For controlling the depletion of neutrophils, blood white cells were also analyzed by staining with PE-anti-mouse Gr-1 and PE-anti-mose Ly6C.

PBMCs were isolated from the blood and stained with FITC-conjugated anti-mouse DX5 and PE-conjugated anti-mouse CD3e, and used for flow cytometric analysis. DX5$^+$CD3$^-$ cells were considered as NK cells.

Assay of cytotoxicity of NK cells

CFSE labeled YAC-1 cells were incubated with splenocytes at the ratio of 1:20 for 6 h. The culture medium containing 50 U/ml IL-2 was used for the incubation of the cells. The

cells were then stained with allophycocyanin-Annexin-V (BD Biosciences, San Diego, CA) and analyzed by flow cytometry. The cytotoxicity was expressed as the percentage of $CFSE^{+}$Annexin-V^{+} cells in $CFSE^{+}$ cells as described.

Immunohistochemistry

Tissue sections were prepared and subjected to immunohistochemical analysis, as described previously (supplementary reference 1). Rabbit anti-mouse DX5 was used as the primary antibody for detecting NK cells. HRP-conjugated Goat anti-Rabbit IgG was used as the secondary antibody. Images were obtained using an Olympus-IX71 microscope at 40 × 10 magnification. The NK cells were counted using Image-Pro Plus 6.0 software. The NK density was defined as the number of NK cells per five randomly chosen high-power fields.

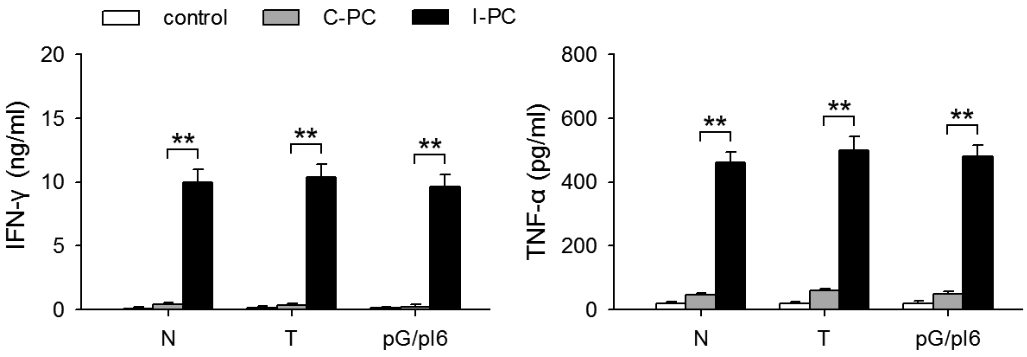

Supplemental Figure S1: IFN-γ and TNF-α in C-PC and I-PC of different mice. The peritoneal cavity of mouse was rinsed with 5 ml of PBS. After centrifugation, the concentration of IFN-γ and TNF-α in the supernatant was detected by ELISA. Data are pooled from four independent experiments with a total of eight samples in each group. **$p < 0.01$.

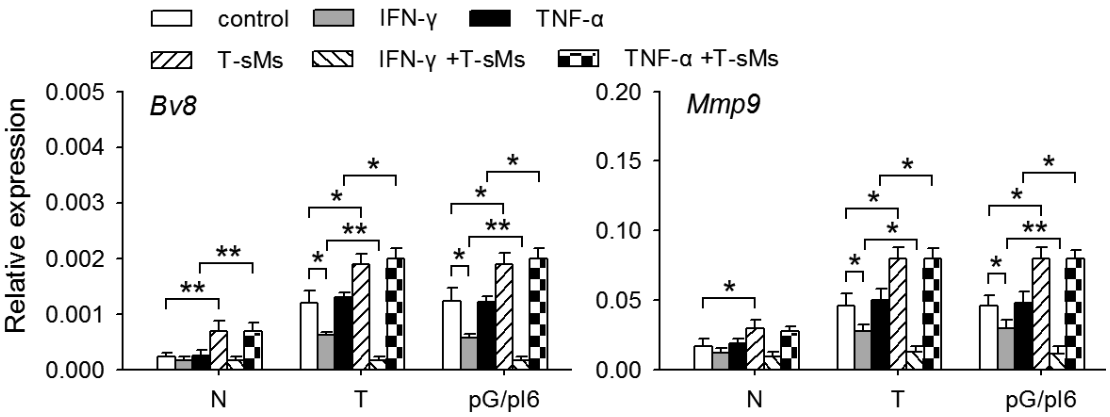

Supplemental Figure S2: Effect of IFN-γ and TNF-α on the expression of *Bv8* and *Mmp9* in neutrophils. Neutrophils were isolated from the C-PC of naive mice, tumor-bearing mice and pG/pI6-mice. The neutrophils were treated with IFN-γ (20 ng/ml) or TNF-α (10 U/ml) for 6 h, and then stimulated with T-sMs for 12 h. The expression of *Bv8* and *Mmp9* was detected by real-time RT-PCR. Data are pooled from four independent experiments with a total of eight samples in each group. *$p < 0.05$, **$p < 0.01$.

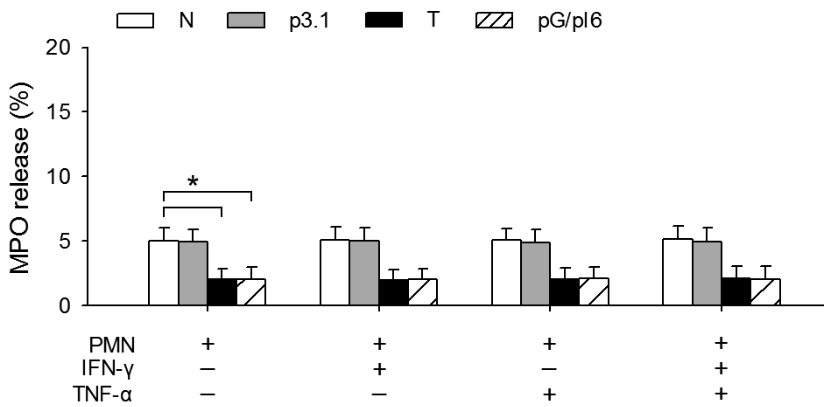

Supplemental Figure S3: IFN-γ and TNF-α do not increase the spontaneous degranulation of neutrophils. Neutrophils were isolated from the C-PC of naive mice, tumor-bearing mice, and pG/pI6-mice, and then treated with IFN-γ (20 ng/ml) and/or TNF-α (10 U/ml) for 6 h. MPO release was detected. Data are pooled from four independent experiments with a total of eight samples in each group. *$p < 0.05$.

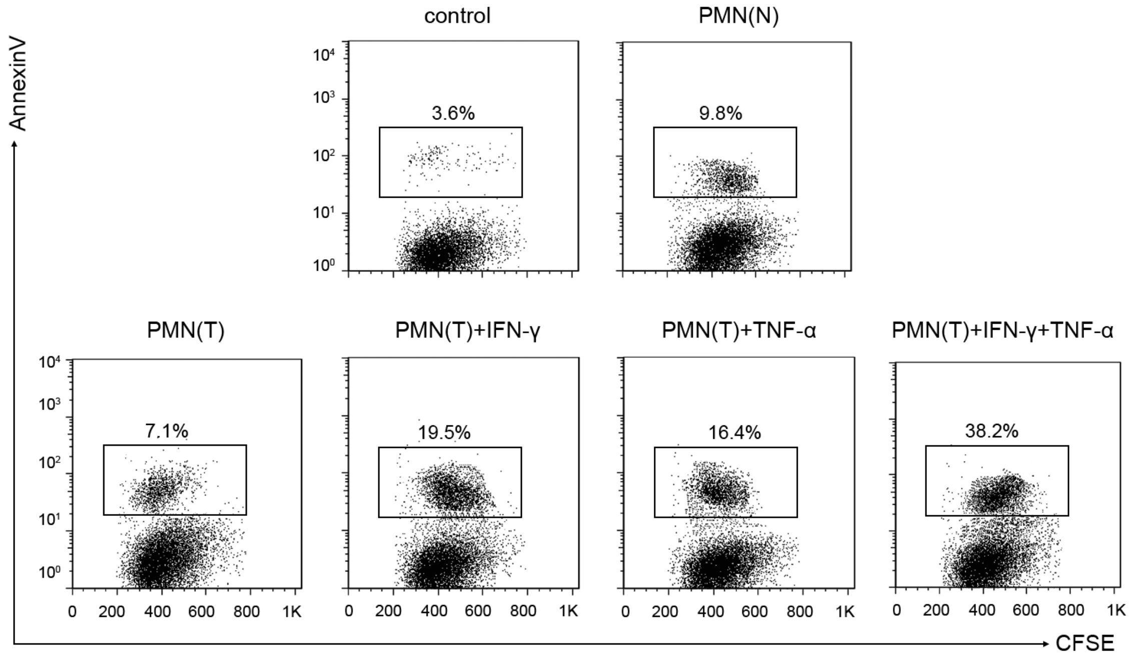

Supplemental Figure S4: IFN-γ and TNF-α augment the cytotoxicity of neutrophils to tumor cells. The cytotoxicity of neutrophils to H22 tumor cells was detected as described in Methods. The representative flow cytometry tracings of H22 cells are shown.

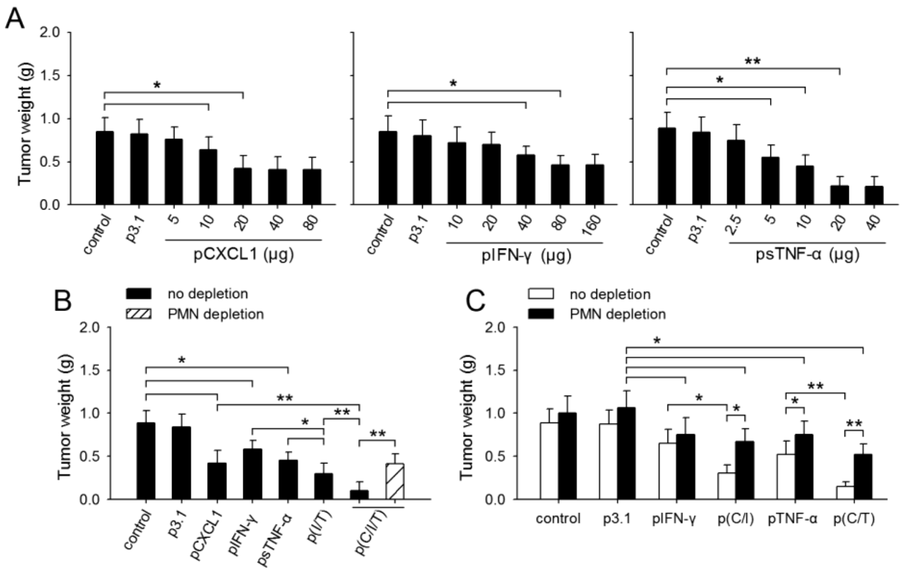

Supplemental Figure S5: Inhibitory effect of local gene transfection on tumor growth. (A) Local transfection of expression vectors pIFN-γ and psTNF-α suppresses tumor growth in a dose-dependent manner in naive mice. Naive mice were inoculated with H22 cells. The mice were treated with plasmids pCXCL1, pIFN-γ, or psTNF-α at the indicated dosage as described in Methods. Tumors were dissected and weighed on d11 after inoculation. **(B and C)** Naive mice were inoculated with H22 cells. The mice were treated with plasmids pCXCL1 (20 μg), pIFN-γ (40 μg) and psTNF-α (10 μg). Neutrophils were depleted (PMN depletion) as indicated. Tumors were dissected and weighed on d11 after inoculation. Data are pooled from four independent experiments with a total of eight samples in each group, **p* < 0.05, ***p* < 0.01.

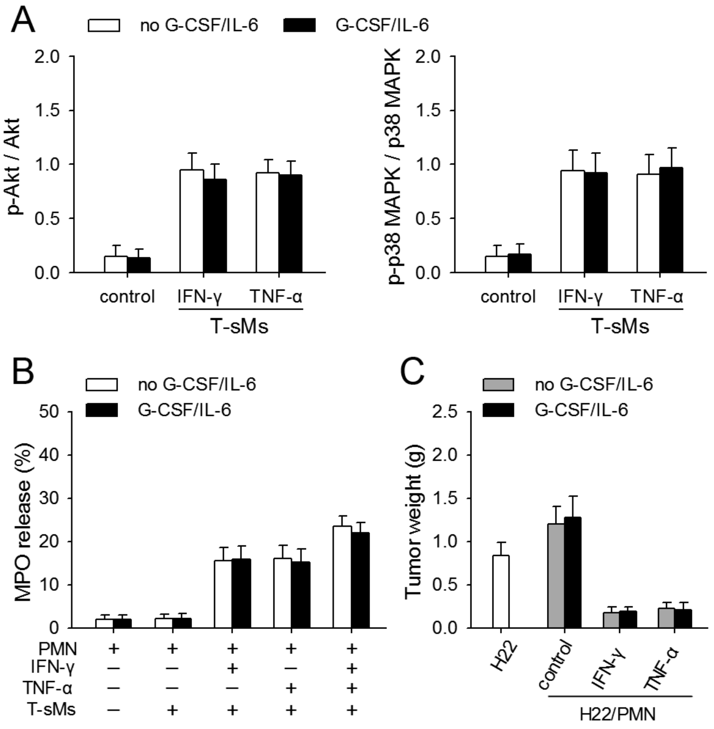

Supplemental Figure S6: G-CSF/IL-6 can not antagonize the priming of neutrophils by IFN-γ and TNF-α in vitro. Neutrophils were isolated from C-PC of pG/pI6-mice. The neutrophils were untreated or treated with IFN-γ (20 ng/ml) or TNF-α (10 U/ml) in absence or presence of G-CSF (50 ng/ml)/IL-6 (50 ng/ml) for 6 h (A-C). Then, the neutrophils were stimulated with T-sMs (0.5 mg/ml) for 0.5 h (A, B) or without further stimulation (C), and used in following experiments: **(A)** The phospho-Akt, Akt, phospho-p38 MAPK, and p38 MAPK were detected by Western blot. The ratios of phospho-Akt to Akt (p-Akt/Akt) and phospho-p38 MAPK to p38 MAPK (p-p38 MAPK/p38 MAPK) were calculated after densitometric analysis. **(B)** MPO release was detected. **(C)** The neutrophils were used for co-inoculation with H22 to naive mice. Tumors were dissected and weighed on d11 after inoculation. Data are pooled from four independent experiments with a total of eight samples in each group (a-c).

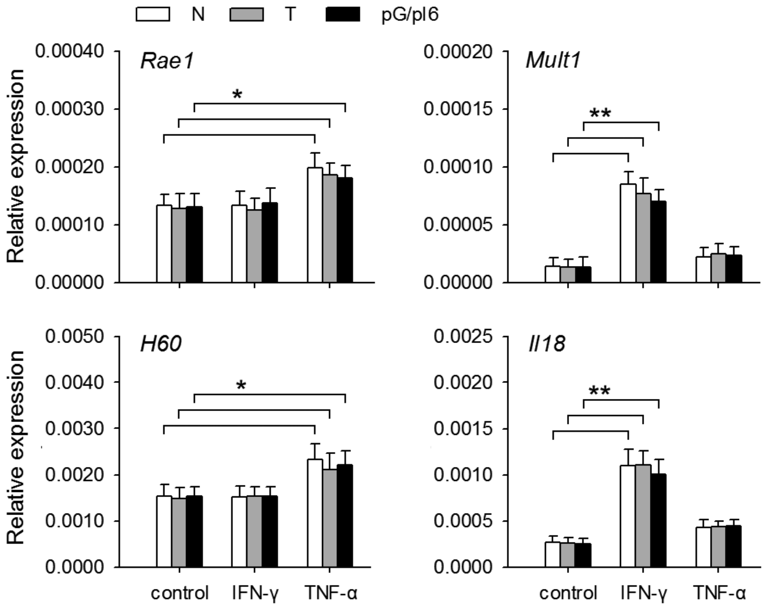

Supplemental Figure S7: IFN-γ and TNF-α augment the NK activating capability of neutrophils. Neutrophils were isolated from C-PC of naive mice, tumor-bearing mice and pG/pI6-mice and stimulated with IFN-γ or TNF-α for 12 h. The expression of *Rae1*, *Mult1*, *H60* and *Il18* was detected by real-time RT-PCR. Data are pooled from four independent experiments with a total of eight samples in each group. *$p < 0.05$, **$p < 0.01$.

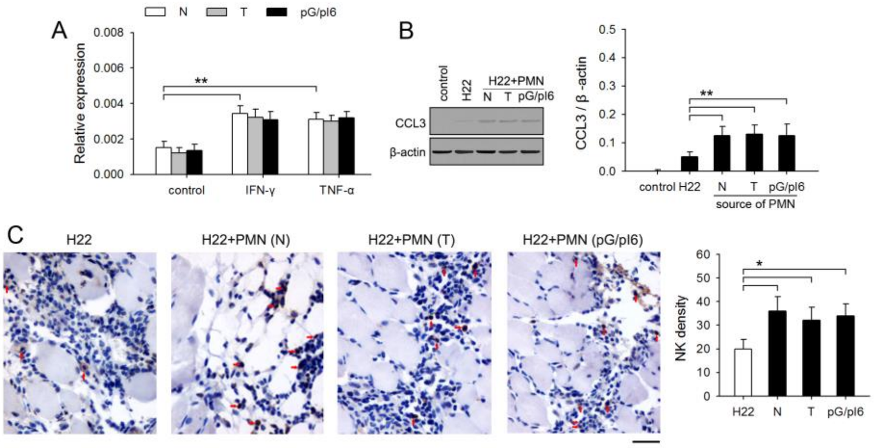

Supplemental Figure S8: Neutrophils could promote the infiltration of NK cells. (**A**) Neutrophils were isolated from C-PC of naive mice, tumor-bearing mice and pG/pI6-mice. The neutrophils were stimulated with IFN-γ (20 ng/ml) or TNF-α (10 U/ml) for 12 h. The expression of *Ccl3* gene was detected at the mRNA level by real-time RT-PCR. (**B**) Neutrophils isolated from I-PC of naive mice, tumor-bearing mice and pG/pI6-mice were used for co-inoculation with H22 to naive mice. CCL3 in the tissue at inoculation site was detected by Western blot 72 h after inoculation (B, *left*). The ratios of CCL3/β-actin were calculated after densitometric analysis of Western blots (B, *right*). **(C)** Neutrophils were isolated from I-PC of naive mice, tumor-bearing mice and pG/pI6-mice. The neutrophils were used for co-inoculation with H22 cells to naive mice. NK cell infiltration was detected by immunohistochemistry 72 h after inoculation (Bar, 25 μm) (left). NK density was calculated (right). Data are representative of three independent experiments (B, left; C, left), or pooled from four independent experiments with a total of eight samples in each group (A; B, right; C, right). *$p < 0.05$, **$p < 0.01$.

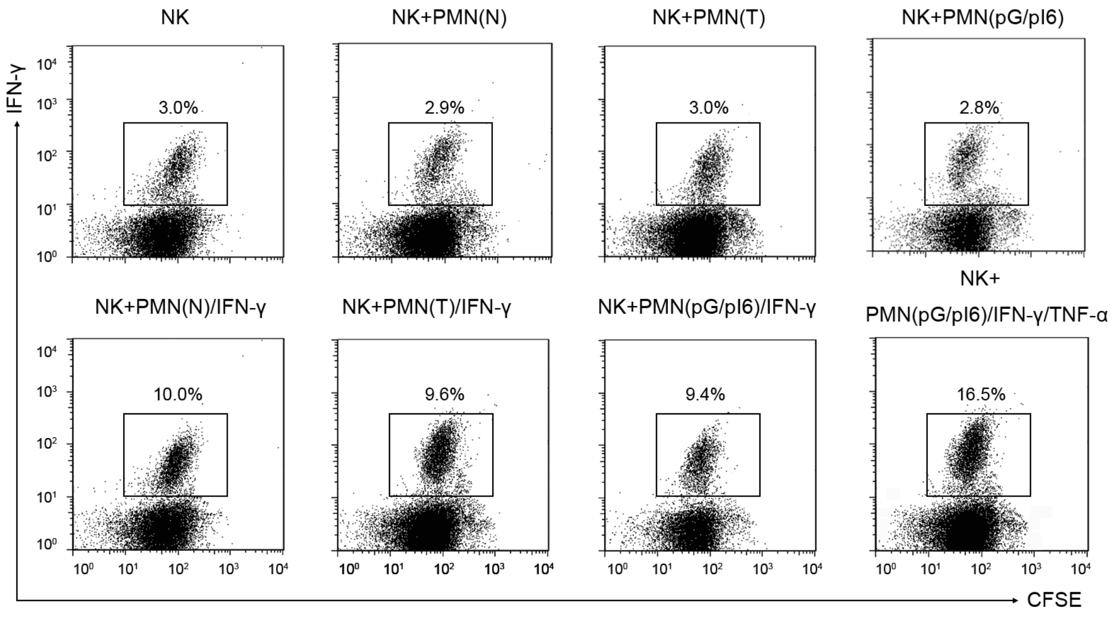

Supplemental Figure S9: IFN-γ and TNF-α augment the NK-activating capability of neutrophils. Neutrophils were isolated from C-PC of naive mice, tumor-bearing mice and pG/pI6-mice. After the treatment with IFN-γ (20μg/ml) and/or TNF-α (10 U/ml) for 12 h, and the removal of IFN-γ and TNF-α by washing with PBS, the neutrophils were co-cultured with the NK cells isolated from naive mice for 12 h. IFN-γ-expressing NK cells were detected by flow cytometry as described in Methods. The representative flow cytometry tracings of NK cells are shown.

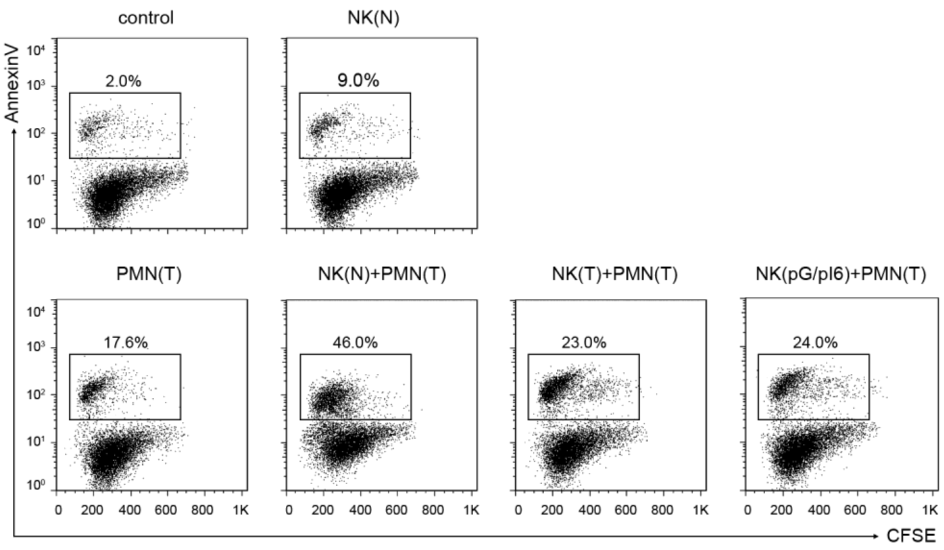

Supplemental Figure S10: The primed neutrophils only cooperate with the NK cells from naive mice to produce stronger cytotoxicity to H22. Neutrophils were isolated from I-PC of tumor-bearing mice. NK cells were isolated from spleen of naive mice, tumor-bearing mice and pG/pI6 mice. Neutrophils (2×10^6) and NK cells (1×10^5) were co-cultured for 12 h, and then incubated with H22 cells (1×10^5) for the assay of cytotoxicity as described in Methods. The representative flow cytometry tracings of H22 cells are shown.

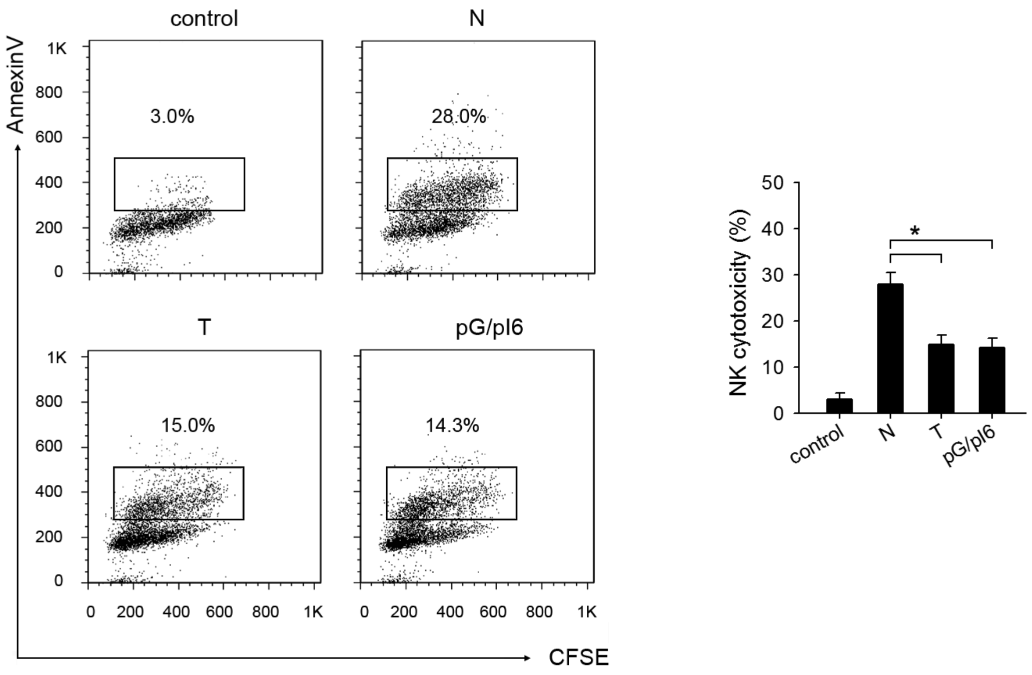

Supplemental Figure S11: Antitumor capacity of NK cells in tumor-bearing mice and pG/pI6-mice is impaired. Splenocytes were isolated from naive mice, tumor-bearing mice, and pG/pI6-mice. CFSE labeled YAC-1 cells were incubated with splenocytes at the ratio of 1:20 for 6 h. The culture medium containing 50 U/ml IL-2 was used for the incubation of the cells. The cells were then stained with allophycocyanin-Annexin V (BD Biosciences, San Diego, CA) and analyzed by flow cytometry. Data are the representative of, or pooled from, four independent experiments with a total of eight samples in each group. *$p < 0.05$.

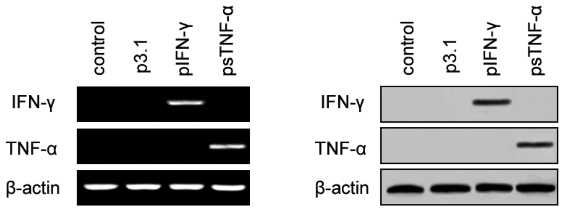

Supplemental Figure S12: Identification of expression vectors by in vivo transfection. Mice received i.m. injection of plasmid DNA in the hind thigh. pIFN-γ (100 μg per mouse) or psTNF-α (50 μg per mouse) was injected. Saline (control) and pcDNA3.1 plasmid (p3.1) were used as controls. The tissues at the sites of injection were surgically excised 48 h or 72 h later, and homogenized. In vivo expression of the vectors was detected at mRNA level by RT-PCR (48 h) and protein level by Western blot (72 h). Data are representative of three independent experiments.

Note: To detect the expression of vectors, total RNA was extracted from tissues with TRIzol reagent. Conventional RT-PCR was used to detect the mRNA produced by expression vector. The primer sequences were as follows: *Actb*, sense 5’-ATCTCCTGCTCGAAGTCT AGAG-3’, antisense 5’-ATGGGTCAGAAGGACTCCTATG-3’; mouse *Ifng*, sense 5’-AAG TGGCATAGATGTGGAA-3’, antisense 5’-TGGCTGGCAACTAGAAG-3’; mouse *Tnf*, sense 5’-GTCGTAGCAAACCACCAAG-3’, antisense 5’-CAGTGGGAGTGGCACCTT-3’. Antisense primer is complementary to the sequence of expression vector.

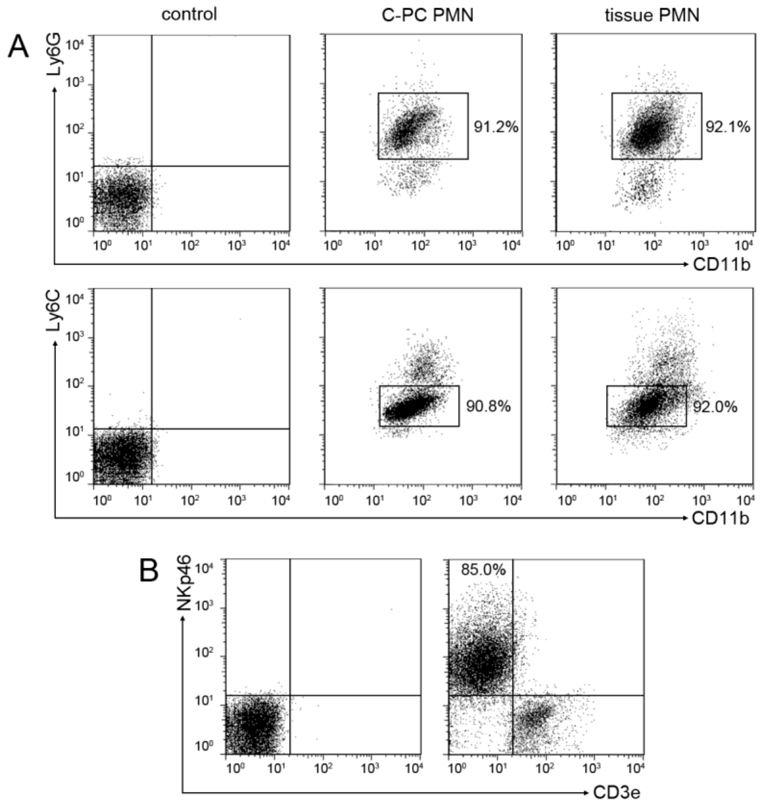

Supplemental Figure S13: Analysis of the isolated neutrophils and NK cells. (A) Neutrophils were isolated from C-PC or tissues as described in Methods. The cells were stained with PE-Cy7-conjugated anti-mouse CD11b and PE-conjugated anti-mouse Ly6G or PE-conjugated anti-mouse Ly6C, and used for flow cytometric analysis. **(B)** NK cells were isolated from spleen cells using anti-DX5 magnetic microbeads and MiniMACS columns. The cells were stained with FITC-conjugated anti-CD335 (NKp46) and PE-conjugated anti-mouse CD3e, and used for flow cytometric analysis. Data are representative of three independent experiments.

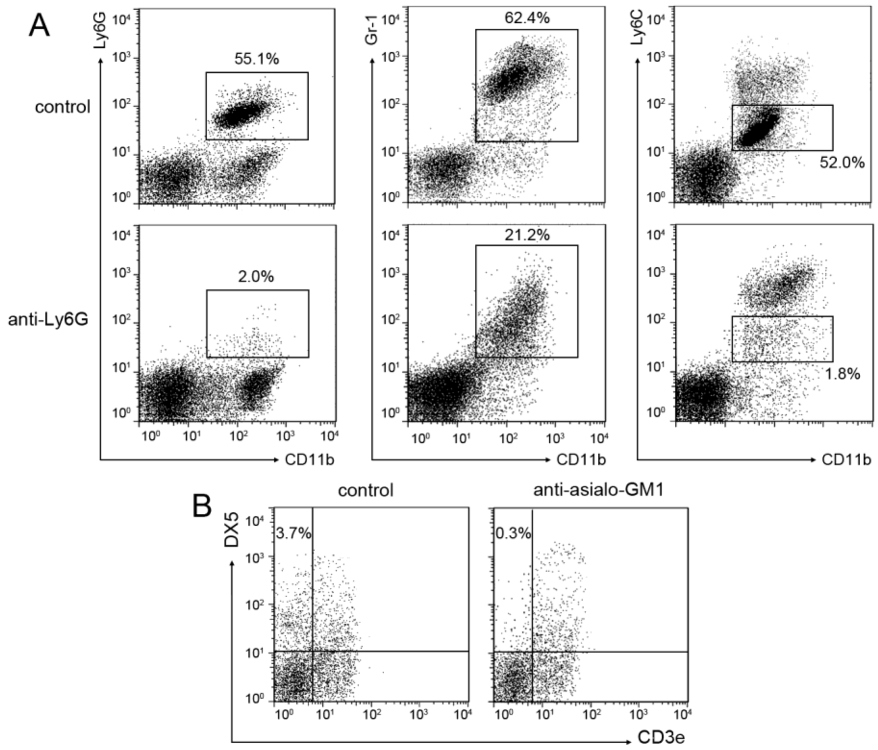

Supplemental Figure S14: Analysis of neutrophil depletion and NK cell depletion in vivo. (A) Neutrophils were depleted in vivo as described in Methods. The neutrophils ($Ly6G^{+}CD11b^{+}$) in blood were detected by flow cytometry one day after the second injection of the antibody. Blood white cells were also analyzed by staining with anti-Gr-1 antibody and anti-Ly6C antibody. **(B)** NK cells were depleted in vivo as described in Methods. The NK cells in PBMC were detected by flow cytometry one day after the second injection of the antibody. The representative flow cytometry tracings are shown.

Supplemental Reference

1. Geng H, Zhang GM, Li D, Zhang H, Yuan Y, Zhu HG, Xiao H, Han LF, Feng ZH. Soluble form of T cell Ig mucin 3 is an inhibitory molecule in T cell-mediated immune response. J Immunol 2006;176:1411-20.
